# Utilization of Long-Lasting Insecticide Treated Nets and Parasitaemia at 6 Months after a Mass Distribution Exercise among Households in Mbarara Municipality, Uganda: A Cross-Sectional Community Based Study

**DOI:** 10.1155/2018/4387506

**Published:** 2018-08-01

**Authors:** Simpson Nuwamanya, Noel Kansiime, Emmanuel Aheebwe, Cecilia Akatukwasa, Harriet Nabulo, Eleanor Turyakira, Francis Bajunirwe

**Affiliations:** ^1^Mbarara University of Science and Technology, Department of Community Health, P.O. Box 1410, Mbarara, Uganda; ^2^Infectious Diseases Research Collaboration, P.O. Box 7475, Kampala, Uganda; ^3^Mbarara University of Science and Technology, Department of Nursing, P.O. Box 1410, Mbarara, Uganda

## Abstract

**Background:**

Utilization of long-lasting insecticide treated nets (LLINs) after free and mass distribution exercise has not been adequately studied. The objectives of this study were to assess ownership and utilization of LLINs following a mass distribution campaign in a Ugandan urban municipality.

**Methods:**

We conducted a cross-sectional study in western Uganda among households with children under 5 years, at 6 months after a mass LLIN distribution exercise. We administered a questionnaire to measure LLIN ownership and utilization. We also measured parasitaemia among children under five years.

**Results:**

Of the 346 households enrolled, 342 (98.8%) still owned all the LLINs. LLIN use was reported among 315 (91.1%) adult respondents and among 318 (91.9%) children under five. Parasitaemia was detected among 10 (2.9%) children under five. Males (OR=2.65, 95% CI 0.99-7.07), single respondents (OR=10.35, 95% CI 1.64-65.46), having a fitting bed net size (OR= 3.59, 95% CI 1.71-7.59), and no childhood malaria episode reported in the home in the last 12 months (OR=1.69, 95% CI 1.02-2.83) were all associated with LLIN use.

**Conclusions:**

Ownership of LLIN is very high, and parasitaemia among the children was very low. Low parasitaemia may be attributed to high LLIN utilization. Long term follow-up should be done to determine durability of the ownership and utilization.

## 1. Background 

Malaria is among the major causes of morbidity and mortality, and young children under five years and pregnant women are particularly at high risk. About half of the world's population live in countries endemic or at risk for malaria transmission.[1] The World Health Organization (WHO) estimated that 212 million cases of malaria occurred globally in 2015 with 627,000 deaths. Africa accounted for 90% of the cases and 92% of the deaths, and over 75% of the deaths were among children under 5 years of age [2].

At health facilities in sub-Saharan Africa, between 30 and 40% of all fevers seen in health centres are due to malaria with huge seasonal variability between rainy and dry seasons.[3] Among the 6 countries with the highest burden of malaria in the WHO Africa region, Uganda is ranked fourth [4]. Malaria accounts for 25-40% of all outpatient visits at healthcare facilities [5] in Uganda. Also, up to 20% of all hospital admissions and 15% of inpatient deaths are due to malaria in this country.

Millions of lives can be saved from malaria episodes and deaths if proven interventions such as long-lasting insecticide treated nets (LLINs) to prevent malaria are made universally available [6]. Several randomized controlled studies have demonstrated the efficacy of LLINs in the prevention of malaria [7–9]. As a result, malaria control programs in endemic countries have made LLINs a key component of their control. In Uganda, the Ministry of Health through the National Malaria Control Program, recently distributed LLINs countrywide in fulfilment of one of the strategies to control malaria transmission. One LLIN was given for every two individuals in a household at no cost to them.

The evaluation of LLIN utilization following mass distribution has presented a mixed picture. While many programs have reported successful utilization following mass distribution [10–12], there is concern that LLINs distributed for free in mass exercises may not always be used for the purpose that was intended. Some studies have shown that LLINs that are distributed for free end being abused [9, 13, 14]. One study in Uganda showed households were using the distributed LLINs to dry fish in the fishing villages along Lake Victoria [15]. More studies are needed to evaluate ownership and utilization of the nets following these mass distribution exercises. Such studies will inform malaria control programs on how best to conduct subsequent distributions. Therefore, the aim of this study was to measure utilization of bed nets following a mass distribution exercise and willingness to purchase new nets and to evaluate effectiveness of LLINs among children under 5 years, using malaria parasitaemia as a measure of effectiveness.

## 2. Methods and Materials 

### 2.1. Study Area

We conducted a cross-sectional study in Mbarara municipality, of western Uganda, a periurban setting in western Uganda. The municipality has three divisions of Kamukuzi, Kakoba, and Nyamitanga. The last two are the most populous in the municipality. Kakoba has 21 villages and a population of about 34,689, and about 60% of the municipality residents live there [16]. Nyamitanga division has 16 villages and a population of about 17,272. Kamukuzi division is the least populated and is an upscale neighborhood of Mbarara; hence this division was left out in the sampling. The economy of Mbarara municipality is predominantly based on small scale businesses, social services sector, and trade. Mbarara is the largest urban setting in south western Uganda and a transit town on the highway to Rwanda and the Democratic Republic of Congo [17].

### 2.2. Data Collection

Between the months of December 2013 and March 2014, all households in the municipality received each at least two LLINs of two different colors, white and blue, as part of a government supported program to control malaria. The community based cross-sectional study was conducted between August to end of September 2014, the beginning of a rainy and moderate transmission period. The study participants were household heads or any other persons aged 18 years and above found at home and able to answer the questions. The household was eligible to participate in the survey if they had at least one child under five and had received a LLIN in the distribution exercise.

We administered a semistructured questionnaire and asked questions on demographics, socioeconomic status, ownership of the distributed LLIN, utilization, and reasons for not using for those who reported nonuse.

### 2.3. Sample Size

According to the Uganda Demographic and Health Survey report of 2011 [18], 53.3% of under- fives in urban areas sleep under a bed net, and parasitaemia in this age group is 30.5%. We made the assumption that mass distribution of free bed nets increased coverage of bed net use and will lead to significant reduction in parasitaemia in under-five children.

We used the Fleiss formula [19] for sample size estimation with continuity correction and adjusting for 10% nonresponse rate, taking into account the design effect arising from selecting households within the same village. We assumed a design effect of 1.2 in the adjustment of sample size. After the adjustment, we estimated that a sample of 324 households with at least one child under five years were sufficient to estimate ownership. The survey included only those households that had at least one child under five. For each household, we recruited one child in the eligible age bracket.

### 2.4. Testing for Malaria

We tested the children under five years for parasitaemia during the household survey and used a malaria rapid diagnostic test (MRDT) HRP2 (pf) [20, 21] that was 98% sensitive and 97% specific to detect* P. falciparum* parasites. Consent to draw blood from the children was obtained from their parents/guardian. Malaria test results were disclosed to the household head or any other guardian. The children that tested positive for malaria were referred to the nearby health facility for care. A laboratory technician conducted the malaria tests.

### 2.5. Inclusion and Exclusion Criteria

Households were eligible to participate if they were located in the two selected divisions, received LLINs in the recent mass distribution net campaign by Ministry of Health, and had at least one child under the age of five years. The distribution list of LLINs was obtained from the local village council chairperson in each village. The household head or any household member aged at least 18 years present at home at the time of the survey was requested to provide informed consent. We excluded households that did not receive LLINs or did not have children under 5 years or where the household representative was not willing to consent or was mentally handicapped.

### 2.6. Quality Control

The study tools were translated from English to the local language of* Runyankole-Rukiga* and then back translated into English to check for accuracy of translation. Questionnaires were pretested in a division outside the study area. Findings from the pilot exercise were used to revise the questionnaire to improve on the systematic setting of the questions. Research assistants were trained on completion of study tools and malaria rapid testing procedures, and finally all the completed questionnaires were checked for completeness and accuracy and stored safely after each field day. Data entry was done by a qualified data officer who used a pretested and validated SQL database.

### 2.7. Sampling Procedure and Data Collection

A multistage sampling procedure was used at three levels, namely, division (or subcounty), parish, and village. From each division, one parish was randomly selected from the sampling frame of parishes. At the parish level, the villages were stratified into either predominantly rural or urban. Within each stratum, one village was randomly selected to obtain two villages from each parish. Overall, the four villages were selected from the two divisions. We conducted the study in the two largest divisions in the municipality because these not only were representative of the majority of inhabitants in the municipality, but also would provide the required number of participants conveniently. The division that was left out in this study is an upscale neighborhood, is sparsely populated, and therefore does not represent the majority of the inhabitants in the municipality.

We obtained the lists of parishes and households that benefited from the mass bed net distribution exercise from the division headquarters, local village chairpersons, and village health team members (VHTs), respectively. Parish and village household lists formed the sampling frame. Each parish contributed to the sample size in proportionate to the size of its population. The consent process for the household head or representative was done at the time of the interview. No prior notification was given to the households about the date of the interview. The interviews were conducted by trained research assistants using semistructured questionnaires.

### 2.8. Outcome Measurements

The primary outcome for this study was LLIN use. LLIN utilization was defined as sleeping under a mosquito net that was received from the mass distribution exercise in the night before the survey. The secondary outcome was ownership, and this was defined as having all the LLINs that were received from the mass distribution. Our study also measured willingness to pay for another net and parasitaemia among under-five children.

### 2.9. Data Analysis Plan

Data analysis was done by use of STATA version 12.0 (Stata Corp., College station, Texas, USA) to estimate the proportion of bed net ownership, utilization, and willingness to purchase new nets and the 95% confidence intervals for these proportions. Logistic regression was used to determine the odds ratios for the factors influencing utilization of LLINs and those associated with malaria parasitaemia among children less than five years. Bivariate analysis was done to evaluate associations between several independent factors and LLINS use among households as the outcome. We calculated crude odds ratios (cOR) and their 95% CI.

To identify factors independently associated with use of LLINs, we entered variables that were found significant in the bivariate analysis (p ≤ 0.05) into a multivariable regression analysis using a stepwise manner. Variables that did not improve the fit of the regression model as measured by log likelihood test were removed. We used the final model to obtain the adjusted odds ratios (aOR) for the association between various factors and LLIN use. We report the aOR and the 95% CI.

### 2.10. Human Subject Issues

The approvals to conduct the study were obtained from the Faculty of Medicine Research Committee and Mbarara University of Science and Technology Research Ethics Committee; subsequently, the study was registered with Uganda National Council for Science and Technology (UNCST). Informed consent was obtained from study participants. Confidentiality and privacy of participants were maintained by use of identification numbers instead of names on data collection sheets. Study participants were told that malaria testing and treatment were available at the nearby health facilities in case they needed it.

## 3. Results

### 3.1. Demographic and Economic Characteristics of Respondents

We enrolled 346 households with children under five years of age that had received LLINs in the recent mass distribution exercise. Majority of respondents were female (92%) (Table 1), with the mean age in years of 29.4 (standard deviation=8.2). Almost 50% had attained primary level of education and 8% did not have formal education. Business was the main source of income for households among 172 (50%) respondents. The median size of the household was 4 (IQR=3-5). Additional social demographic and economic characteristics of respondents are shown in Table 1.

### 3.2. Ownership and Use of LLINs

Almost 99% (342/346) of the households in the study area still owned the LLINs that were received from the mass distribution exercise. The median number of nets in the household was 2 (IQR=2-3). However, a large majority (300/346 or 86.7%) had at least one bed net before the mass distribution exercise.

About 4.3% (15/346) of the respondents mentioned they had given the net to the relatives whom they thought needed the bed nets more than them, and about 3 (0.8%) nets were sold to obtain money to purchase other household items. Majority of the households (183 or 52.8%) received white bed nets. Majority of the participants, 304 (87.8%), reported that the size of bed nets which were received from the mass distribution fitted their sleeping facility.

Over 90% (315 of 346) reported that all members in their households had slept under a mosquito bed net the night before the interview. Among children under five, 91.9% (315/346) of the households reported that under-fives had slept under LLINs the night preceding the survey and that 83.7% (289 of 346) of children aged five years and above had slept under a mosquito net.

### 3.3. Parasitaemia among Children under 5 Years

We tested all children under five years for malaria using rapid tests, and 10 (2.9%) of the 346 children tested positive for malaria. Parasitemia did not differ by prior ownership of LLINs. We did not test the adults.

### 3.4. Reasons for Not Sleeping under the Bed Net

Among persons who did not sleep under the bed net in the night prior to the survey (n=31), some mentioned lack of sufficient space to hang the net, 11 (35.4%); perceived poor quality of the net, 23 (74.1%); not having enough bed nets 23 (74.1%); being allergic to the bed nets 4 (12.9%); and, for one respondent, the land lord refusing to hang the net.

### 3.5. Challenges with Using the LLINs

Participants were asked whether they had experienced any challenges in hanging the nets; for the adults, the following were mentioned: allergy like reactions (n=8), not balancing (n=7), lack of sufficient space (n=11), no hook to attach (n=6), no bed (n=1), and net not fitting (n=6) and being too rough (n=2). Among the children, the reasons given for children who did not use an LLIN were as follows: not having enough bed nets (n=), forgetting to put up the net (n=1), too hot weather (n=5), net being wet (n=2), net being owned by the father (n=1), lack of enough space (n=6), land lord refusing to hang the net (n=1), net being too rough (n=2), and net being worn out (n=2).

### 3.6. Bivariate Analysis for Factors Associated with Bed Net Use

The results for the bivariate analysis for factors associated with bed net use are presented in Table 2. The odds of using an LLIN among males were 2.8 times more than among the females** (**OR = 2.8, 95% CI**:** 1.10, 7.09, p=0.02). Marital status was significantly related to the use of an LLIN. The single respondents were 10.3 times more likely to use bed nets OR=10.35 (95% CI 1.64, 65.46) compared to the married ones. Also, using the married respondents as a referent category, those who were divorced or separated were 3.1 times more likely to use bed nets (OR=3.1 % CI: 1.49, 20.03).

Having a net that fits on the sleeping facility in the household was associated with a 3.97 increase in the odds of using it compared to the respondents whose nets were not fitting on the sleeping facility (OR=3.97 95% CI: 1.96, 8.07, p= <0.001). Respondents who had challenges in hanging were less likely to use the LLIN compared to those who did not experience challenges while hanging bed nets, but this association was marginally significant (OR=0.59 95% CI: 0.35, 1.01, p=0.053), and these results are shown in Table 2. Finally households that had not had a child suffering from malaria in the last 12 months were 1.86 times more likely to use bed nets compared to respondents who reported a child having suffered from malaria in the last 12 months (OR= 1.86 95% 1.15, 2.99 p= 0.011).

### 3.7. Multivariable Analysis for Factors Independently Associated with Bed Net Use

The results of the multivariable analysis for factors independently associated with bed net use are presented in Table 3. These factors included gender of the respondents, male (aOR=2.65 95%CI: 0.99, 7.07; p = 0.051); marital status of the respondents especially if a respondent was single (aOR = 10.35 95% CI: 1.64, 65.46 p= 0.013) or widowed (aOR = 5.08 95% CI: 1.03-25.16; p = 0.047) compared to the married ones; reported fitting size of the bed net in the house (aOR = 3.59 95%CI: 1.71, 7.59; p= 0.001); number of bed nets in the household (aOR = 2.49 95% CI: 1.56, 3.99; p = <0.001) and having no child who suffered from malaria in the last 12 months ( aOR=1.69 95% CI: 1.02, 2.83; p= 0.043). The odds of bed net use were 1.69 times higher among households reporting that no children had suffered in the last 12 months compared to those that reported having a child that suffered from malaria in the last 12 months.

## 4. Discussion

Our study reports a near universal LLIN ownership and utilization after mass bed net distribution exercise in a periurban area of western Uganda. This level of bed net ownership is higher than what has been reported elsewhere in Uganda [22–24]. Our findings also show higher ownership than in other studies outside Uganda [13, 25, 26]. However the high levels of ownership and utilization are not unique in our study and are comparable to those seen in other African settings where similar mass distribution exercises were conducted in Equatorial Guinea [27], Madagascar [10], and Ethiopia [28].

Our data show a very high proportion of both ownership and utilization of the LLINs. Many malaria control programs in sub-Saharan Africa distribute LLINs free-of-charge, and there has been concern that, because the nets are free, some households may not value them and hence not use them. A study in Rwanda [29] showed exactly this. There was a high level of LLIN ownership but lower levels of utilization following a mass distribution. In this Rwandan study, males and families from a lower socioeconomic group were less likely to use the LLINs, but our results differ from the Rwandan study.

In our study, only a small proportion of the households did not have all the nets that were received from the mass bed net distribution exercise. These households reported they had sold them for money to buy household items and food or disposed them off because of being torn beyond repair, and others gave the nets to their relatives because they thought the relatives needed them more. This may have been done because some respondents believed that they would receive new replacement nets free-of-charge through a similar mass distribution campaign as has been documented before [15, 30, 31].

According to a recent study in western Uganda [32], the high proportion of bed net use may be attributed to appreciation of the benefits of LLIN utilization and perceived threat from malaria as a major health problem. Malaria control programs need to ensure this high utilization is sustained through continuous sensitization. Our study shows that a high proportion of study participants were willing to purchase new LLINs after the free nets wore off. This proportion is higher than what was reported in a similar study in Ethiopia [24].

There were several factors that were significantly associated with utilization of an LLIN. Households that reported utilization were also less likely to report a malaria episode in the past 6 months. The positive outcomes may strengthen and reinforce the behavior to sleep under a net. However, our cross-sectional design was not sufficiently strong to demonstrate a cause-effect relationship between utilization and reports of a malaria episode. Respondents who were single were more likely to utilize LLINs compared to those who were married. Marital status is important for LLIN use because our data show that being single was associated with a 10.4-fold increase in the odds of using a bed net compared to being married or cohabiting. The findings of the relationship between marital status and LLIN use are in agreement with what was reported in a study conducted from Cameroon [33]. The reasons for this finding were not explored in this study. It is difficult to explain these findings, and further examination is necessary especially in study designs using qualitative methodology to understand the ramifications of these findings to gain in-depth knowledge about them.

The households that owned bed nets which fitted on the sleeping facility were more likely to use bed nets compared to their counterparts. This is important for future distribution exercises that sizes of nets distributed should conform to the nature of the houses in the distribution area. Many study participants live in smaller two-roomed houses, and hence the size of the nets does matter. Majority of the participants received rectangular bed nets and reported these as being easy to hang.

Our data show that having three or more bed nets in the household was strongly associated with bed net use. This finding may be explained in three ways. First, the more nets exist in a household, the more likely that everyone gets a net to sleep under. Second, the mass distribution exercise only provided two or three nets per household; therefore some households already had a net even prior to the distribution exercise. The households that pre-owned a net are therefore more likely to have more nets after the exercise and also more likely to use them. Third, households that already had a net are also more motivated to use nets and therefore a distribution exercise may reinforce the utilization.

Our data show that households that had no children under five suffering from malaria in the last 12 months were more likely to use bed nets. The finding indicates the protective nature of the LLIN. Our study was not primarily designed or even powered to demonstrate the effectiveness of the LLINs in prevention of malaria, since this is well known from randomized trials [8, 9, 34]. Our data, though observational, point to the effectiveness of LLINs. The finding is important as it reinforces use of LLIN among households, as they will likely attribute the absence of malaria in the households to the utilization of LLINs.

In this study we found out that the parasitaemia among children under five is low at 2.9% of the 346 children that were screened for malaria parasites. This parasitaemia is much lower than expected based on recent results of the nationwide malaria indicator survey [35] which showed that parasitemia was 19.1%. The low parasitaemia in our study may be attributed to consistent use of bed nets among this age group or the ‘herd immunity' acquired from the large scale availability of the long-lasting insecticide treated bed nets in the community. This level of parasitemia is comparable to what was reported in a study conducted among children in the same age bracket for over six years in south western Uganda, which reported 3 percent in the urban areas [36]. Given that this survey was done long before mass distribution of LLINs in Mbarara, it is likely that the low parasitaemia may not be wholly attributed to the current mass LLIN use. Other factors such as wide scale use of ACTs may be responsible [37].

Our study has important strengths. First, we conducted a community based study using a random sampling approach to obtain an unbiased sample to represent the municipality. Second, the sample size was large enough. Our study also has some limitations. First, we were not able to determine level of parasitaemia in blood as we only had access and funding to have RDTs. Measurement of parasitaemia would have detected clinically significant infection. We required microscopy in order to measure parasitaemia and this was not available to us. Additionally, given their increased vulnerability to malaria, only the children under 5 were tested and not the adults due to limited funding available for this study. Second, the study used a quantitative design which does not permit exploration of attitudes, experiences, and practices associated with bed net use in the households. Lastly, the study used a cross-sectional study design which does not define the temporal relationship of the independent and dependent variables. For instance, we did not have data on parasitaemia prior to the use of bed net. Our study has important weaknesses too. First, we did not collect qualitative data to report the reasons for the high level of ownership and utilization. Second, the history of malaria episodes wqw self-reported and some of these may not have been true malaria episodes. And lastly, our follow-up time of 6 months is short.

At policy level, we recommend that routine mass bed net distribution campaigns should continue. A survey on the sleeping facilities, shape of the bed net, and size of the bed net should be conducted before the bed net mass distribution exercise so that what is distributed is appropriate and fits within the sleeping facilities of the recipients.

Our data shows that a significant proportion of the respondents were willing to purchase new bed net if and when the nets from the free distribution became worn out. This is an important finding for the sustainability of LLIN use. The initial concern that free mass distribution may create complacency for future use of LLINs is unfounded. However, governments can support social marketing groups to make the LLINs available at subsidized rates. We made our evaluation at 6 months, but longer term follow-up is necessary to measure the durability of this high bed net ownership and utilization.

In conclusion, ownership of bed nets is very high after 6 months following a mass distribution exercise. Parasitaemia among children under five is very low and may be partly attributed to use of LLINs. Programs will need to develop strategies to prevent sale or handover of these nets to third parties to sustain long term ownership. Long term follow-up needs to be done to determine durability of the ownership.

## Figures and Tables

**Table 1:** Sociodemographic and economic characteristics of the respondents (n=346).

**Characteristics**	**n (**%**)**
**Place of residence**	
Kakoba division	191 (55.2)
Nyamitanga division	155 (44.8)
**Median household size **	4 [IQR=3-5]
**Median number of children ≤5 in the household **	1 [IQR=1-2]
**Gender of the respondents**	
Male	27 (7.8)
Female	319 (92.2)
**Age of the respondent**	
<25	100 (28.9)
25-34	165 (47.7)
35 or more	81 (23.4)
**Religion of the household head**	
Catholic	106 (30.6)
Protestant	133 (38.4)
Muslim	73 (21.1)
Others	34 (9. 8)
**Marital status **	
Single	26 (7. 5)
Married	290 (83.8)
Widowed	11 (3. 2)
Others	19 (5. 5)
**Education Level of the respondents**	
No formal education	28 (8. 1)
Primary	167(48.3)
Secondary	105 (30.4)
Tertiary/University	46 (13.3)
**Occupation of the respondents**	
Unemployed	33 (9. 5)
Business	172 (49.7)
Professional	30 (8.7)
Domestic services	76 (22.0)
Peasant	22 (6.4)
Other	13 (3.8)
**Number of children under five years**	
1	200 (57.8)
2	116 (33.5)
3 or +	30 (8.7)
**Type of house**	
Permanent	304 (87.9)
Temporary	42 (12.1)
**Median monthly income (USD) of household**	27.5 (IQR 8.3, 55.5)
**Possession of a radio**	
Yes	235 (67.9)
No	111(32.1)
**Possession of Television **	
Yes	163 (47.1)
No	183 (52.9)
**Owned a bed net before mass distribution**	
**Yes**	300 (86.7)
**No**	46 (14.3)
**Possession of mobile phone **	
Yes	293 (84.7)
No	53 (15.3)

**Table 2:** Bivariate analysis of factors associated with utilization of LLINs, Mbarara municipality, Uganda.

**Variable**	**Use a bed net** **n (**%**)**	**Do not use bed net** **n (**%**)**	**cOR (95**%**) CI**	**p-value**
**Division**				
Nyamitanga	88 (56.77)	67 (43.23)	1.00	0.834
Kakoba	110 (57.89)	80 (42.1)	0.96 (0.62-1.47)	
**Gender**				
Female	177 (55.66)	141 (44.34)	1.00	**0.026**
Male	21 (77.78)	6 (22.22)	2.78 (1.10-7.09)	
**Age of respondents**				
<25	58 (58.00)	42 (42.00)	1.00	0.428
25-34	89 (54.27)	75 (45.73)	0.86 (0.52-1.41)	
35 or +	51 (62.96)	30 (37.04)	1.23(0.68-2.24)	
**Marital Status**				
Married/Cohabiting	167 (57.21)	122 (42.21)	1.00	**0.008** **∗**
Single	20 (76.92)	6 (23.08)	10.35 (1.64-65.46)	
Widowed	2(18.18)	9 (81.82)	5.08 (1.03-25.16)	
Separated/ Divorced	9 (47.37)	10 (52.63)	3.12 (1.49-20.03))	
**Education Level**				
No formal education	14 (50.00)	14 (50.00)	1.00	0.469
Primary	91 (54.49)	76 (45.51)	1.20 (0.54-2.67)	
Secondary	65 (62.50)	39 (37.50)	1.67 (0.72-3.86)	
Tertiary	28 (60.87)	18 (39.13)	1.56 (0.60-4.02)	
**Religion **				0.541
Catholic	66 (62.26)	40 (37.74)	1.00	
Protestant	70 (53.03)	62 (46.97)	0.68 (0.41-1.15)	
Muslim	43(58.90)	30 (41.10)	0.87 (0.47-1.59)	
Others	19(55.88)	15 (44.12)	0.77 (0.35-1.68)	
**Number of children <5 in the house**				0.093
3 or +	13 (43.33)	17 (56.67)	1.00	
1	123 (61.81)	76 (38.19)	2.12 (0.97-4.60)	
2	62 (53.45)	54 (46.55)	1.50 (0.67-3.37)	
**Type of house structure**				
Temporary	23 (54.76)	19 (45.24)	1.00	0.713
Permanent	175(57.76)	128 (42.24)	1.13 (0.59-2.16)	
**Possession of a television**				
**No**	105 (57.69)	77 (42.31)	1.00	0.905
**Yes**	93 (57.06)	70 (42.94)	0.97 (0.64-1.49)	
**Possession of a mobile phone**				
No	29 (54.72)	24 (45.28)	1.00	0.669
Yes	169 (57.88)	123 (42.12)	1.14 (0.63-2.05)	
**Number of LLINs in the household**				
3 or less	66 (43.7)	85 (56.6)	1.00	**<0.001** **∗**
More than 3	132 (68.0)	62 (31.9)	2.74(1.76-4.26)	
**Bed net size fitting on the sleeping facility**				
No	12 (28.57)	30 (71.43)	1.00	**<0.001** **∗**
Yes	186 (61.39)	117(38.61)	3.97 (1.96-8.07)	
**Challenges hanging the bed net**				
No	165 (60.00)	110 (40.00)	1.00	0.0532
Yes	33 (47.14)	37 (52.86)	0.59 (0.35-1.01)	
**Has no child in the household who suffered from malaria in the last 12 months**				
No	154 (61.60)	96(38.40)	1.00	**0.011** **∗**
Yes	44 (46.32)	51(53.68)	1.86 (1.15-2.99)	

**Table 3:** Multivariable analysis of factors associated with bed net use, Mbarara municipality, Uganda.

**Variable**	**aOR (95**%** CI) **	**p-value**
**Gender**		
Female	1.00	**0.051**
Male	2.65 (0.99-7.07**)**	
**Marital status**		
Married/ Cohabiting	1.00	
Single	10.35 (1.64-65.46)	**0.047** *∗*
Widowed	5.08 (1.03-25.16)	
Divorced/ separated	3.12 (1.49-20.03)	
**Bed net size fitting on the sleeping facility **		
No	1.00	
Yes	3.59 (1.71-7.59)	**0.001** **∗**
**Number of Bed nets in the Household**		
3 or less	1.0	**<0.001** **∗**
More than 3	2.49 (1.56-3.99)	
**Has no child with malaria in the past 12 months**		
No	1.0	**0.043** **∗**
Yes	1.69 (1.02-2.83)	

*∗*Significant at 0.05 level.

## Data Availability

On request, the authors will be able to provide all the data and materials that were obtained or used during the conduct of this research study.
